# Editorial: The Impact of Systems Medicine on Human Health and Disease

**DOI:** 10.3389/fphys.2016.00552

**Published:** 2016-11-24

**Authors:** Adil Mardinoglu, Jens Nielsen

**Affiliations:** ^1^Department of Biology and Biological Engineering, Chalmers University of TechnologyGothenburg, Sweden; ^2^Science for Life Laboratory, KTH–Royal Institute of TechnologyStockholm, Sweden

**Keywords:** systems medicine, metabolic networks and pathways, systems biology, networks medicine, biological networks, metabolic diseases

Complex disorders including obesity, diabetes, fatty liver diseases, cardiovascular disease (CVD), and cancer are results from a combination of genetic, environmental, and lifestyle factors. The prevalence of such disorders has increased dramatically in the last two decades and there is an urgent need for the development of new prognostic tools for the treatment of such diseases. However, this requires a deep understanding of the underlying molecular mechanisms involved in the occurrence of the diseases. With the advances in high throughput technologies, biological components of cells can be measured with a very high resolution and these data can be used for investigating whole systems properties using a network-based approach. Systems medicine provides an integrative platform for studying the interactions between the biological components of the cell using a holistic approach and generating mechanistic explanations for the emergent systems properties (Mardinoglu and Nielsen, 2012). This inter-disciplinary field of study allows for understanding biological processes of cells in health and disease states, gaining new insights into what drives the appearance of the disease, and finally identifying proteins and metabolites implicated in human disease. Systems medicine utilizes mathematical approaches to generate models which can be employed for designing new sets of experiments and for mapping the response of the system to perturbations quantitatively. These models as well as the developed tools can accelerate the emergence of personalized medicine which can transform the practice of medicine and offer better targets for drug development with minimum side effects.

Genome Scale Metabolic Models (GEMs) are important tools in systems biology and employed for simulating the metabolism of cells/tissues in different states (Mardinoglu et al., 2013). GEM includes all known metabolic reactions and associated protein coding genes in a particular cell or tissue and the reconstruction of GEMs is costly and time consuming process. Pacheco et al. benchmarked the algorithms that have been used in the automated reconstruction of context-specific GEMs. This may allow researchers to identify limitations of each method and help them to increase the quality of context-specific reconstruction algorithms and as well as the generated models. Zhang and Hua reviewed the potential applications of GEMs in systems medicine and industrial biotechnology. The authors described the key concepts and assumptions used in the application of the GEMs and provided detailed explanation about the recent applications which may promote the use of GEMs by biologists.

GEMs have been widely used in the discovery of biomarkers and drug targets that can be used in the development of efficient treatment strategies (Mardinoglu and Nielsen, 2015). These integrative models can also be used in the stratification of the patients as well as the identification of the subjects at risk of developing complex diseases. CVD includes all the diseases of the heart and circulation and continues to constitute the leading cause of death globally. Therefore, there is an urgent need to develop tools and methods for identifying individuals at risk of developing CVD. Björnson et al. reviewed the current CVD risk scores and discussed how systems medicine could improve the identification of risk and maximize personalized treatment benefit.

Tumor cells alter their metabolism to maintain their growth and proliferation. Ghaffari et al. reviewed the metabolic alterations that are known to occur in cancer and underlined the use of genome-scale metabolic modeling approach in perceiving a system level perspective of cancer metabolism which can be used in discovery of biomarkers and drug targets. Özcan and Çakır have reconstructed glioblastoma multiforme (GBM) specific GEMs based on gene expression data and used their context-specific model to predict metabolic flux distributions in the brain tumor cells. The authors identified the GBM specific metabolic alterations and provided a comprehensive coverage of tumor metabolism. Moreover, flux distributions in glycolysis, glutaminolysis, TCA cycle, and lipid metabolism were predicted and validated by additional computational analysis and literature information.

Zhang et al. evaluated the potential value of current major systems biology-based approaches, including genome wide association studies, gene regulatory networks, and protein–protein interaction networks and GEMs. The authors discussed how integrative analysis of personal multi-omics data may provide increased understanding of personal genotype–phenotype relationships. Moreover, the potential benefit of integrating different type of networks in increased understanding of the interactome is discussed. Recently, metabolic networks have been integrated with other biological networks and these networks have been used in the analysis if omics data obtained from different clinical conditions (Lee et al., 2016a,b). In the same context, Brown reviewed the challenges in the generation of SuperModels where computational models were integrated with various data types and analytics. The author has also discussed how these SuperModels may assist in the development of personalized and precision medicine.

Stable isotope assisted metabolomics techniques have been used in systems medicine for metabolic flux analysis and pathway discovery. It is essential to understand the pathophysiology of dyslipoproteinemia in humans since it has major role in the progression of complex diseases including CVD and diabetes. The use of tracers labeled with stable isotopes and mathematical modeling may provide a powerful tool for probing lipid and lipoprotein kinetics *in vivo*. Adiels et al. reviewed the recent kinetic studies and discussed how these studies may improve our understanding of impaired human lipoprotein metabolism. Even though, stable isotope labeling analyses have been highly targeted, in recent years, tools for the global non-targeted detection, quantification, and computational analysis of isotopic enrichment have become available. Weindl et al. discussed how such non-targeted stable isotope labeling analyses can be applied for systems medicine applications.

Finally, Shmelkov et al. described an approach to improve *in silico* identification of a comprehensive ensemble of targets for any drug weighted by the expression of those receptors in relevant tissues. Their approach may increase the sensitivity of target detection and allow for systematic integration of bioactivity/docking scores between drugs/compounds and proteins with the expression patterns of those proteins in human tissues.

The work presented in this Research Topic addresses many of the recent progress in the applications of the GEMs is medical applications. The integration of GEMs with other biological networks and generation of whole cell models may foster the development of personalized medicine which may increase benefits and reduce risks for patients.

## Author contributions

AM and JN wrote the editorial together and approved its final version.

### Conflict of interest statement

The authors declare that the research was conducted in the absence of any commercial or financial relationships that could be construed as a potential conflict of interest.
